# Different subpopulations of macrophages, neutrophils, mast cells, and fibroblasts are involved in the control of tumor angiogenesis

**DOI:** 10.3389/fmed.2024.1481609

**Published:** 2024-10-08

**Authors:** Domenico Ribatti

**Affiliations:** Department of Translational Biomedicine and Neuroscience, University of Bari Medical School, Bari, Italy

**Keywords:** angiogenesis, fibroblasts, macrophages, mast cells, neutrophils, tumor progression

## Abstract

The tumor microenvironment comprises diverse cell types, including T and B lymphocytes, macrophages, dendritic cells, natural killer cells, myeloid-derived suppressor cells, neutrophils, eosinophils, mast cells, and fibroblasts. Cells in the tumor microenvironment can be either tumor-suppressive or tumor-supporting cells. In this review article, we analyze the double role played by tumor macrophages, tumor neutrophils, tumor mast cells, and tumor fibroblasts, in promoting angiogenesis during tumor progression. Different strategies to target the tumor microenvironment have been developed in this context, including the depletion of tumor-supporting cells, or their “re-education” as tumor-suppressor cells.

## Introduction

Tumor cells undergo a Darwinian selection and can survive and enter an equilibrium state where the innate and adaptive immune system controls the tumor (1, 2). Some tumor cells acquire mutations, chromosome amplifications and deletions, and epigenetic modifications, resulting in gene silencing or synthesis of abnormal proteins. Overall, these events allow tumor cells to escape the control of the immune system, increase, and give rise to a clinically detectable tumor.

The link between chronic inflammation and tumorigenesis was first proposed by Rudolf Virchow in 1863 after the observation that infiltrating leukocytes are a hallmark of tumors and first established a causative connection between the lymphoreticular infiltrate at sites of chronic inflammation and the development of cancer (3). Dvorak described tumors as wounds that never heal (4). Under a variety of inflammatory conditions, both innate and adaptive immune cells are capable of polarization into their “tumoricidal” (growth arresting) or “tumorigenic” (growth promoting) forms. The tumor microenvironment comprises diverse cell types, including T and B lymphocytes, macrophages, dendritic cells, natural killer cells, myeloid-derived suppressor cells, neutrophils, eosinophils, mast cells, and fibroblasts. Cells in the tumor microenvironment can be either tumor-suppressive or tumor-supporting cells. Different strategies to target the tumor microenvironment have been developed in this context, including the depletion of tumor-supporting cells, or their “re-education” as tumor-suppressor cells. In this review article, we analyze the double role played by tumor macrophages, neutrophils, mast cells, and fibroblasts, in promoting angiogenesis during tumor progression.

## Macrophages

Two different macrophage subpopulations have been described: classically activated or inflammatory macrophages (M1) and alternatively activated or anti-inflammatory macrophages (M2) (Table 1). M2 macrophages can be divided into four subsets consisting of M2a, M2b, M2c, and M2d, based on the stimuli used to derive them in tissue culture experiments (5). M1 macrophages are induced by interferon-gamma (IFNγ), tumor necrosis factor-alpha (TNFα), or lipopolysaccharide (LPS), and secrete different pro-inflammatory cytokines, including TNFα, interleukin 1 alpha, beta, 6, 12, 23 (IL-1α, IL-1β, IL-6, IL-12, IL-23), cyclooxygenase 2 (COX-2), whereas M2 macrophages are induced by IL-4, IL-10, IL-13, IL-21, IL-33, activin A, corticosteroids, prostaglandins (PGs), and vitamin D3 (6).

**Table 1:** Comparative differences between M1 and M2 macrophages.

Pro-inflammatory macrophage (M1)
Classically activated
Polarized by lipopolysaccharide (LPS) and interferon-gamma (IFNγ)
Releases immunostimulatory cytokines
Tumor suppressing phenotype
Releases anti-angiogenic factors
Phagocytes tumor cells
Stimulates T helper cells
Anti-inflammatory macrophages (M2)
Alternatively activated
Polarized by interleukin-4 and − 13 (IL-4 and IL-13)
Release anti-inflammatory cytokines
Release pro-angiogenic factors
Promote metastasis
Impede anti-tumor response from T cells

Macrophages are the most represented immune cells in the tumor microenvironment. Tumor-associated macrophages (TAMs) include tissue-resident macrophages (TRMs) and a large proportion of bone marrow-derived macrophages (BMDMs). TRMs derive from CX3CR1^+^ Kit^+^ erythromyeloid progenitors, while BMDMs originate from peripheral blood mononuclear cells. TRMs, including alveolar macrophages in the lung, brain microglia, and Kupffer cells in the liver, develop in the embryonic yolk sac and fetal liver, self-maintain throughout adulthood, and are involved in tissue homeostasis and integrity (7). BMDMs derive from circulating monocytes that exit the bloodstream and undergo differentiation into macrophages within different tissues. Monocyte chemoattractant protein-1 (MCP-1) secreted by activated fibroblasts, endothelial cells, vascular smooth muscle cells, monocytes, and T cells, triggers chemotaxis and migration of monocytes by interacting with the CC chemokine receptor 2 (CCR-2) on monocytes (8). TRMs and BMDMs are involved in forming a pre-metastatic niche, facilitating cancer engraftment at the metastatic sites (9).

TAM infiltration correlates with angiogenesis, poor prognosis, tumor progression, and metastasis in different tumors, including ovarian and breast cancer, follicular B lymphoma, soft tissue sarcoma, classic Hodgkin lymphoma, melanoma, glioma, squamous cell carcinoma of the esophagus, and bladder and prostate carcinoma. Increased TAMs are associated with poor prognosis and therapeutic resistance (9, 10). Single-cell RNA sequencing (scRNA-seq) analysis has demonstrated the co-existence of multiple subsets of TAMs in individual tumors, showing that TAMs simultaneously co-express M1 and M2 marker genes (11). TAMs with M1 phenotype suppress tumor formation through direct phagocytosis of tumor cells, the induction of T cell-mediated cell cytotoxicity, and the stimulation of antibody-mediated immune response.

TAMs generally display an M2-like phenotype (12). In the initial stage of cancer, TAMs exert an immunostimulant function. In contrast, at later stages, they acquire an M2 phenotype, exerting a tumor-promoting function, promoting angiogenesis, repairing and remodeling wounded or damaged tissues, and suppressing adaptive immunity (12, 13). M2 TAMs produce immune-suppressive cytokines, including PGE2, IL-10, and transforming growth factor beta (TGFβ) (14). They can also suppress dendritic cell differentiation and inhibit their functions through IL-10 production. The accumulation of M2 TAMs is linked to a poor prognosis in human cancers. The phenotype of polarized M1-M2 TAMs may be reversed (15), and a continuum exists between the two phenotypes (16). Negative regulation of CD47 and its ligand signal regulatory protein alpha (SIRPα) can restore TAM phagocytic capacity (17).

TAMs are generally localized in the hypoxic areas of tumors, where they express hypoxia-inducible factor 1 alpha (HIF1α) that, in turn, induces the transcription of the angiogenic factors vascular endothelial growth factor (VEGF), fibroblast growth factor 2 (FGF-2), and platelet derived growth factor (PDGF) (18). TAMs regulate the angiogenic switch in a mouse model of breast cancer (19). VEGF restores delayed tumor progression in tumors depleted of macrophages (20). Pharmacological depletion of TAMs results in an inhibition of angiogenesis in tumors (12). TAMS express a broad array of angiogenesis-modulating enzymes, including matrix metalloproteinase (MMP)-2, −7, −9, −12, and cyclooxygenase-2 (COX-2) (21–23).

Radiotherapy or chemotherapy increases the number of M2 TAMs favoring tumor recurrence (24, 25). Therapeutic strategies to reduce TAMs pro-tumoral activities include reduced monocyte recruitment, promotion of macrophage phagocytosis, and induction of M2 macrophage reprogramming, which may be obtained with different strategies including receptor tyrosine kinase RON inhibitors, angiopoietin-2 (Ang-2) receptor inhibitors, histone acetyl deacetylase (HDAC) inhibitors, PI3kδ inhibitors, miRNA inhibitors, CD40 agonists, Toll-like receptor (TLR) agonists, and macrophage receptors with collagenous structure (MARCO) neutralization antibodies.

The primary population of pro-angiogenic TAMs corresponds to TIE-2 expressing monocytes (TEMs), which secrete VEGF and MMP-9 (26). Most of the circulating TEMs do not express endothelial cell/endothelial precursor cells markers, such as VEGFR-2, AC133, CD146, and CD34, whereas they express hematopoietic markers, such as CD45. Moreover, circulating human TEMs do not express CCR-2, the receptor for MCP-1, a chemokine that regulates the recruitment of monocytes to inflamed tissues and tumors. TEMs might be attracted to tumors in a CCR-2-independent manner, by signals produced by tumor cells, stromal cells, or endothelial cells. TEM knockout prevents human glioma neovascularization in a mouse model and induces tumor regression (26). Ang-2 (a TIE-2 ligand) blockade abrogates TIE-2 expression and inhibits tumor growth and metastasis by impairing angiogenesis (27). TEMs do not differentiate into endothelial cells, suggesting that their pro-angiogenic activity could consist of a paracrine stimulation of angiogenesis. TEMs are localized both in perivascular and avascular viable (hypoxic) areas of tumors and are absent in non-neoplastic tissues adjacent to tumors (28). Exposure to both hypoxia and Ang-2 markedly suppressed the release of an anti-angiogenic IL-12 (29).

The selective elimination of TEMs using a suicide gene impaired angiogenesis in mouse tumors and induced substantial tumor regression and TEM elimination does not affect the overall number of TAMs and granulocytes, indicating that TEMs represent a distinct monocyte subset with specific pro-angiogenic activity (26, 29).

## Neutrophils

Neutrophils are the most abundant circulating leukocytes, constituting a significant component of infiltrated immune and inflammatory cells in the tumor microenvironment. Besides their recruitment to primary tumors, neutrophils accumulate in the blood and distant organs of tumor-bearing hosts. Tumor-associated neutrophils (TANs) are polarized in anti-tumor (N1) or pro-tumor (N2) phenotypes. N1 TANs are short-living, highly cytotoxic, and highly immune-stimulating. They recruit and activate immune cells by producing cytokines, chemokines, and proteases able to stimulate T cell proliferation, NK, and dendritic cell maturation (30, 31). N2 TANs are long-living, low-cytotoxic, with high pro-angiogenic, pro-metastatic, and immunosuppressive activities (32, 33). In mouse tumor models, TANs assume N1 or N2 phenotype and function, according to different tumor progression times. TGFβ stimulates N2 and inhibits N1 polarization, whereas inhibition of TGFβ results in a shift to the N1 phenotype (32). N2 TANs release different angiogenic factors, such as VEGF, IL-8, TNF-*α*, hepatocyte growth factor (HGF) and MMPs (34–36). Microarray analysis has demonstrated about thirty angiogenesis-relevant genes in human neutrophils (37). Neutrophil contribution to pathological angiogenesis may be sustained by an autocrine amplification mechanism.

VEGF release occurs at sites of neutrophil accumulation. Production and release of VEGF from neutrophils depend on granulocyte-colony stimulating factor (G-CSF) (38). Moreover, neutrophil-derived VEGF can stimulate neutrophil migration (39).

## Mast cells

Mast cells are well known for their role in allergies and autoimmunity, but they can also infiltrate tumors, where exert both pro- and anti-tumorigenic activities depending on their microenvironmental stimuli. Mast cells attracted in the tumor microenvironment by stem cell factor (SCF) secreted by tumor cells produce several angiogenic factors as well as MMPs, which promote tumor vascularization and invasiveness, respectively (40). H1 receptor antagonists significantly improved overall survival rates and suppressed tumor growth as well as the infiltration of mast cells and VEGF levels through the inhibition of HIF-1*α* expression in B16F10 melanoma-bearing mice (41). Mast cells exert immunosuppression releasing TNF-α and IL-10 and stimulating immune tolerance and tumor promotion (42, 43). Mast cells may promote inflammation, inhibition of tumor cell growth, and tumor cell apoptosis by releasing cytokines, such as IL-1, IL-4, IL-6, IL-8, monocyte chemotactic protein-3 and -4 (MCP-3 and MCP-4), TGF-*β*, and chymase. Chondroitin sulfate inhibits tumor cell diffusion and tryptase causes tumor cell disruption and inflammation through the activation of protease-activated receptors (PAR-1 and -2) (44).

Mast cells store in their secretory granules pre-formed active serine proteases, including tryptase and chymase (45). Tryptase stimulates the proliferation of endothelial cells, promotes vascular tube formation *in vitro*, degrades connective tissue matrix, and activates MMPs and plasminogen activator, which in turn degrade the extracellular matrix with consequent release of VEGF or FGF-2 from their matrix-bound state (46). Mast cells contain MMPs, and tissue inhibitors of MMPs (TIMPs), which intervene in regulation of extracellular matrix degradation, allowing the release of angiogenic factors. Mast cell-deficient W/Wv mice exhibit a decreased rate of tumor angiogenesis (47). Development of squamous cell carcinoma in a human papillomavirus (HPV) 16 infected transgenic mouse model of epithelial carcinogenesis provided experimental support for the early participation of mast cells in tumor growth and angiogenesis (48, 49). Mast cells infiltrated hyperplasia, dysplasias, and the invasive front of carcinomas, but not the core of tumors. Accumulation occurred proximal to developing capillaries and the stroma surrounding the advancing tumor mass (48). Infiltration of mast cells and activation of MMP-9 coincided with the angiogenic switch in premalignant lesions through the release of pro-angiogenic molecules from the extracellular matrix. Remarkably, premalignant angiogenesis was abrogated in a mast cell-deficient HPV 16 transgenic mouse indicating that neoplastic progression in this model involved infiltration of mast cells in the skin (48, 49). An increased number of mast cells have been demonstrated in angiogenesis associated with vascular tumors, like hemangioma and hemangioblastoma, as well as several hematological and solid tumors, including lymphomas, multiple myeloma myelodysplastic syndrome, B-cell chronic lymphocytic leukemia, breast cancer, gastric and colon-rectal cancer, uterine cervix cancer, melanoma, and pulmonary adenocarcinoma, in which mast cell accumulation correlate with increased neovascularization, mast cell VEGF and FGF-2 expression, tumor aggressiveness and poor prognosis (40).

## Fibroblasts

Fibroblasts are interconnected with tumor cells by promoting tumor growth, angiogenesis, and the metastatic process (50). Cancer-associated fibroblasts (CAFs) are characterized by the expression of specific markers and secrete growth factors and angiogenic factors (Table 2). A source of CAFs is represented by the expansion of tissue-resident fibroblasts in the early stages of tumor progression (51, 52). CAFs may also originate from transdifferentiation of myofibroblasts, bone marrow-derived mesenchymal stem cells, stellate cells, and adipocytes (53–56). CAFs modulate tumor growth by secreting: (i) growth factors able to increase tumor cell proliferation and exert an anti-apoptotic activity; (ii) chemotactic factors recruiting other stromal cells, including leukocytes, monocytes/macrophages, and mast cells. CAFs have both pro-tumorigenic and anti-tumorigenic roles. CAFs (type 1 polarized fibroblasts) induce immunosuppression by an increase in Th2 cells, Th17 cells, and Tregs, and are also involved in therapy resistance (57). Co-injection of CAFs with tumor cells resulted in enhanced tumor formation (58). CAFs (type 2 polarized fibroblasts) exert a tumor-promoting function under the influence of growth factors and chemokines. They stimulate cancer cell survival, growth, and invasion, by secreting cytokines, exosomes, and growth factors, contribute to angiogenesis through the release of angiogenic cytokines, including VEGF, TGFβ, IL-6, and TNFα, and activate other immune cells (58). ScRNA-seq of precursor lesions of human pancreatic adenocarcinoma (PDCA) revealed dynamic changes in the composition of CAF subsets during tumor progression (59). The progression of premalignant Barrett’s esophagus to esophageal adenocarcinoma is characterized by increased inflammatory-related gene expression by fibroblasts (60).

**Table 2:** Growth factors and angiogenic factors secreted by tumor-associated fibroblasts.

Interleukin-6 (IL-6)
Insulin-like growth factor (IGF)
Hepatocyte growth factor (HGF)
Fibroblast growth factor-2 (FGF-2)
C-X-C Motif Chemokine Ligand 8 (CXCL-8)/ Interleukin 8 (IL-8)
C-X-C Motif Chemokine Ligand 12 (CXCL-12)

## Therapeutic strategies

VEGF/VEGF receptors (VEGFRs) inhibition represents the most widely used anti-angiogenic strategy, including anti-VEGF and anti-VEGFRs specific antibodies, VEGF decoy receptors (VEGF-TRAP), receptor tyrosine kinase (RTK) inhibitors. An alternative anti-angiogenic strategy is the use of Ang2/Tie2 inhibitors.

Tumor microenvironment cells represent attractive therapeutic strategies (61). Different approaches have been developed to enhance TAMs anti-tumor immune activity, including TAM apoptosis by blocking CSF-1/CSF1-R signaling (62); CSF1-R inhibitors suppress macrophage differentiation toward the M2 phenotype and macrophage-related angiogenesis (63); inhibition of TAM recruitment to tumor microenvironment by blocking CCL2 of CCR2 axis, improving the prognosis (64); increase of TAM-mediated phagocytosis of cancer cells; blocking programmed cell death protein (PD-1)/ programmed cell death ligand-1 (PD-L1) signaling improve phagocytic activity of TAMs (65); reprogramming of TAMs by enhancing their antigen presentation to T cells via CD40 agonists, or by promoting their re-education to anti-tumoral phenotypes (66); Ang2/Tie2 signaling inhibits tumor growth by blocking angiogenesis signals and the immunosuppressive functions of TAMs (63).

Different studies have demonstrated the anti-cancer activity of CAFs, including inhibition of fibroblast activation protein, TGFβ inhibitors, or vitamin S analog Paricalcitol (50).

Strategies explored to inhibit neutrophils include the inhibition of CXC receptors like CXCR2 that are associated with the migration of neutrophils to tumor areas. CXCR1 and CXCR2 inhibitors are currently in clinical development in cancer. Inhibition of the IL-23 and IL-17 axis is another approach, as IL-17 and IL-23 stimulate the expansion of neutrophils mediated by G-CSF (67).

Mast cells might act as a new target for the adjuvant treatment of tumors through the selective inhibition of angiogenesis, tissue remodeling, and tumor-promoting molecules, allowing the secretion of cytotoxic cytokines, and preventing mast cell-mediated immune suppression. Pre-clinical studies using anti-c-kit antibodies, anti-TNF-*α* antibodies, or the mast cells stabilizer disodium cromoglycate (cromolyn) in mouse models have demonstrated promising results (68).

## Concluding remarks

This mini review provides an overview of our knowledge of the crosstalk between different inflammatory cell subpopulations and tumor angiogenesis. Targeting these cells has proven to be a promising strategy for tumor treatment. The binary concept of dividing these cells into two subpopulations with, respectively, pro- and anti-inflammatory activities is too simplistic considering their functional plasticity and the context-dependent nature of their behaviors and functions. These inflammatory cells exist in a wide spectrum of phenotypes driven by tumor-derived signals and tissue-specific microenvironments. Recent new technologies including CRISPR gene editing and single-cell sequencing allow us to understand better how these cells regulate tumor angiogenesis. Moreover, the potential transition between immunosuppressive and immunostimulatory phenotypes should be further investigated in the context of different biomarkers of signaling pathways. Future exploration and characterization of specific subgroups will lead to a new direction for targeted tumor angiogenesis.
